# PIMT/NCOA6IP Deletion in the Mouse Heart Causes Delayed Cardiomyopathy Attributable to Perturbation in Energy Metabolism

**DOI:** 10.3390/ijms19051485

**Published:** 2018-05-16

**Authors:** Yuzhi Jia, Ning Liu, Navin Viswakarma, Ruya Sun, Mathew J. Schipma, Meng Shang, Edward B. Thorp, Yashpal S. Kanwar, Bayar Thimmapaya, Janardan K. Reddy

**Affiliations:** 1 Department of Pathology, Feinberg School of Medicine, Northwestern University, Chicago, IL 60611, USA; y-jia@northwestern.edu (Y.J.); nliu2224@163.com (N.L.); ruya.sun@northwestern.edu (R.S.); ebthorp@northwestern.edu (E.B.T.); y-kanwar@northwestern.edu (Y.S.K.); 2 Department of Surgery, Division of Surgical Oncology, University of Illinois at Chicago, Chicago, IL 60612, USA; navinv@uic.edu; 3 Next Generation Sequencing Core Facility, Feinberg School of Medicine, Northwestern University, Chicago, IL 60611, USA; m-schipma@northwestern.edu; 4 Feinberg Cardiovascular Research Institute and Department of Medicine, Feinberg School of Medicine, Northwestern University, Chicago, IL 60611, USA; meng.shang@northwestern.edu; 5 Department of Microbiology and Immunology, Feinberg School of Medicine, Northwestern University, Chicago, IL 60611, USA

**Keywords:** PIMT/NCOA6IP, PRIP/NCOA6, PPARα, dilated cardiomyopathy, cardiac fibrosis, energy metabolism

## Abstract

PIMT/NCOA6IP, a transcriptional coactivator PRIP/NCOA6 binding protein, enhances nuclear receptor transcriptional activity. Germline disruption of PIMT results in early embryonic lethality due to impairment of development around blastocyst and uterine implantation stages. We now generated mice with Cre-mediated cardiac-specific deletion of PIMT (csPIMT^−/−^) in adult mice. These mice manifest enlargement of heart, with nearly 100% mortality by 7.5 months of age due to dilated cardiomyopathy. Significant reductions in the expression of genes (i) pertaining to mitochondrial respiratory chain complexes I to IV; (ii) calcium cycling cardiac muscle contraction (*Atp2a1*, *Atp2a2*, *Ryr2*); and (iii) nuclear receptor PPAR- regulated genes involved in glucose and fatty acid energy metabolism were found in csPIMT^−/−^ mouse heart. Elevated levels of *Nppa* and *Nppb* mRNAs were noted in csPIMT^−/−^ heart indicative of myocardial damage. These hearts revealed increased reparative fibrosis associated with enhanced expression of *Tgfβ2* and *Ctgf*. Furthermore, cardiac-specific deletion of PIMT in adult mice, using tamoxifen-inducible Cre-approach (TmcsPIMT^−/−^), results in the development of cardiomyopathy. Thus, cumulative evidence suggests that PIMT functions in cardiac energy metabolism by interacting with nuclear receptor coactivators and this property could be useful in the management of heart failure.

## 1. Introduction

The nuclear receptor coactivators, as exemplified by some components of Mediator complex and others such as PRIP/NCOA6, (proliferator-activated receptor (PPAR) interacting protein (PRIP)/Nuclear receptor coactivator 6) participate in the transcriptional activation of specific genes regulated by nuclear receptors and other transcription factors [1,2,3,4,5]. In an effort to understand the role of coactivator PRIP (NCOA6), we previously isolated a PRIP-interacting protein, designated PIMT/NCOA6IP/TGS1 (PRIP-interacting protein with methyltransferase domain (PIMT)/NCOA6-interacting protein (NCOA6IP)/Trimethylguanosine Synthase1 (TGS1)) an RNA binding protein with RNA methyltransferase activity [1]. PIMT is expressed ubiquitously including in liver, kidney and skeletal muscle. The methyltransferase activity of PIMT hypermethylates 2,2,7-trimethylguanosine cap structures of small nuclear RNA (snRNA), and small nucleolar RNA (snoRNA), that are important in RNA splicing [6,7]. PIMT binds to PRIP under in vivo and in vitro conditions and may serve as a bridge to transduce signals from upstream transcription factor-coactivator complex to the Mediator complex to drive RNA polymerase II mediated gene transcription [8]. Thus, available evidence suggests that coactivators PIMT, PRIP and Med1 are important in nuclear receptor PPARα controlled fatty acid β-oxidation [3].

Because heart derives the bulk of its functional energy from fatty acid β-oxidation, we asked whether PIMT is essential for normal cardiac functions and if cardiac-specific ablation of this gene causes dilated cardiomyopathy (DCM) similar to that noted with ablation of coactivators Med1 and PRIP [9,10]. First, we used a mouse model in which cardiomyocyte-specific deletion of PIMT (csPIMT^−/−^) was carried out during late gestational and early postnatal development by intercrossing PIMT^fl/fl^ mice with α-MyHC-Cre transgenic mice [11]. In this csPIMT^−/−^ mouse model, hearts develop lethal DCM between four to eight months after birth. The csPIMT^−/−^ mouse heart showed severe mitochondrial damage, reduced expression of several genes related to energy metabolism, and calcium signaling related cardiac muscle contraction. Some of the essential findings noted in csPIMT^−/−^ mouse were independently confirmed using another mouse model in which cardiac-specific deletion of PIMT in adult mice was accomplished by using tamoxifen-inducible Cre-approach [9]. Many of the cardiac-specific changes noted in csPIMT^−/−^ mice we report here bear resemblance to that reported recently for the cardiac-specific ablation of coactivators *Med1* and *Ncoa6* [9,10]. Collectively, these observations lead us to propose a model in which a protein complex consisting of PIMT, NCOA6, and MED1 (Mediator1) interact with other chromatin modifiers such as p300/CBP to target a common set of transcription factors to regulate metabolic pathways critical for cardiac functions. 

## 2. Results

### 2.1. Generation of Cardiomyocyte-Specific PIMT Heart Knockout Mice

**Figure 1:**
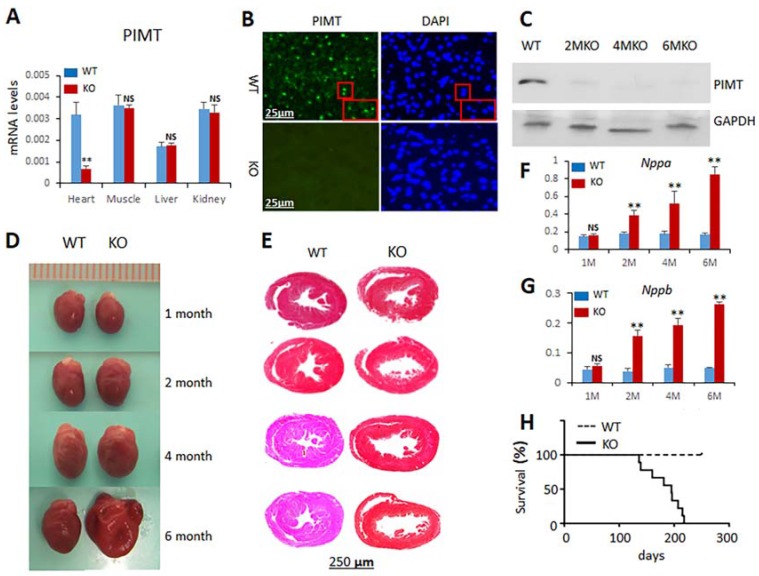
Cardiac-specific ablation of PIMT expression causes dilated cardiomyopathy. (**A**) Quantification of PIMT mRNA relative to 18S ribosomal RNA by RT-qPCR in PIMT^fl/fl^ (WT) and csPIMT^−/−^ (KO) mouse heart, muscle, liver and kidney; (**B**) Immunohistochemical localization of PIMT in 2-month-old PIMT^fl/fl^ (WT) and csPIMT^−/−^ (KO) mouse hearts. Nuclear localization of PIMT is evident in WT but not in KO hearts; compare DAPI stained images shown in right; (**C**) Western blot analysis for detecting PIMT protein level in PIMT^fl/fl^ and csPIMT^−/−^ mouse heart homogenates; (**D**) Representative photographs of heart of 1-, 2-, 4-, and 6-month-old csPIMT^−/−^ mice and their PIMT^fl/fl^ littermate controls. Six-month-old csPIMT^−/−^ mouse hearts were flaccid and flabby; (**E**) Cross sections of hearts shown in Figure 1D were stained with H&E to reveal thinning of ventricular walls and dilation of chambers in csPIMT^−/−^ mouse hearts (right panel); (**F**,**G**) *Nppa* and *Nppb* mRNA levels, respectively, in PIMT^fl/fl^ and csPIMT^−/−^ mouse hearts obtained at indicated ages. Each group was analyzed using 5 different mice (each mouse was assayed separately) and the values were expressed as the mean ± SD. * *p* < 0.05, ** *p* < 0.01, NS: not significant; (**H**) Survival curve showing lethality of mice with csPIMT^−/−^ hearts. 36 mice for each group of PIMT^fl/fl^ and csPIMT^−/−^ were used for the generation of survival curve. Kaplan-Meier method was used to determine the survival rates and data were compared using log rank test. Each group was analyzed using 5 different mice and the values were expressed as the mean ± SD. * *p* < 0.05, ** *p* < 0.01, NS: not significant.

Previously, we reported that global disruption of PIMT gene in mice results in early embryonic lethality by affecting development around blastocyst and uterine implantation stages [11]. To evaluate the heart-specific function of PIMT, we generated mice with cardiomyocyte-specific disruption of PIMT gene (csPIMT^−/−^). We crossed mice with a loxP flanked allele targeting exons 3–4 of PIMT gene (PIMT^fl/fl^) with α-MyHC-Cre recombinase transgenic mice that express Cre-recombinase in cardiomyocytes under the control of α-myosin heavy chain (α-MyHC) gene promoter to yield csPIMT^−/−^ mice following protocols as described in our recent paper [9]. Disruption of the PIMT gene in cardiomyocytes was confirmed by PCR genotyping and by q-PCR analysis of RNA from mouse heart (Figure 1A). PIMT mRNA levels greatly decreased in csPIMT^−/−^ hearts but not in the liver, kidney or skeletal muscle, confirming heart-specific PIMT deletion (Figure 1A). Immunohistochemical localization of PIMT revealed prominent cardiomyocyte nuclear staining in PIMT^fl/fl^ mouse heart but not in the myocardium of csPIMT^−/−^ mouse littermates (Figure 1B). Furthermore, on Western blot analysis, PIMT was barely detectable at the protein level in csPIMT^−/−^ mouse hearts (Figure 1C).

### 2.2. Cardiomyocyte-Specific Disruption of PIMT Causes Dilated Cardiomyopathy in Mice

csPIMT^−/−^ mice are viable at birth with no grossly appreciable morphological abnormalities. There was no significant change in the heart size at two months of age in these csPIMT^−/−^ mice but sectioning revealed mild degree of heart dilation as evidenced by thinning of the walls of left ventricular chamber (Figure 1D,E). The csPIMT^−/−^ mice continued to show myocardial damage, with dilated heart associated with thinning of heart walls. At age six months, csPIMT^−/−^ mice showed significant increase in heart size and they were increasingly flaccid when compared to that of littermate controls (Figure 1E). To further assess the heart damage, we assayed the mRNA levels of heart failure indicators atrial natriuretic peptide (ANP, gene *Nppa*) and brain natriuretic peptide (BNP, gene *Nppb*) [12]. Both *Nppa* and *Nppb* RNA levels increase in heart failure as ventricular cells are recruited to secrete both these peptides in response to left ventricular dysfunction [12]. Both *Nppa* and *Nppb* RNA levels increased dramatically in csPIMT^−/−^ hearts at two months and the levels continued to remain high until six months (Figure 1G,F). Nearly 100% of csPIMT^−/−^ mice died within 7.5 months after weaning due to dilated cardiomyopathy-related atrial and ventricular dilatation and heart failure (Figure 1H).

### 2.3. Echocardiographic Observations of csPIMT^−/−^ Mouse Heart Indicate Poor Contractility

**Figure 2:**
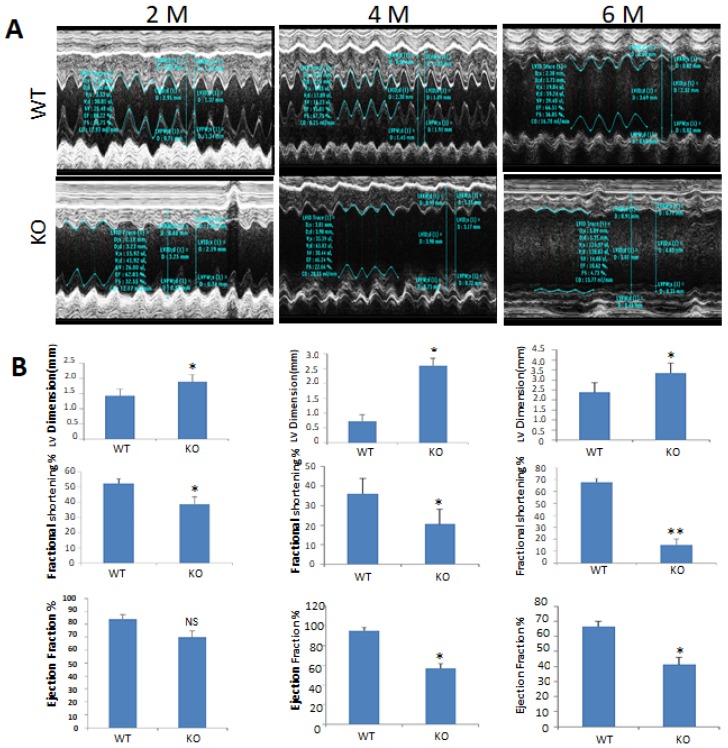
Echocardiographic results showing poor contractility of csPIMT^−/−^ hearts. (**A**) Representative profiles of M-mode echocardiographic analyses of 2-, 4-, and 6-month-old PIMT^fl/fl^ (WT, **upper** panel) and csPIMT^−/−^ (KO, **lower** panel) mice; (**B**) Quantification of left ventricular dimension (upper panel), and fractional shortening (middle panel) and ejection fraction (lower panel) are shown below for 2-, 4-, and 6-month PIMT^fl/fl^ and csPIMT^−/−^ echocardiographic images. Values were expressed as the mean ± SD. * *p* < 0.05, ** *p* < 0.01, NS: not significant.

The effects of PIMT deletion on cardiac function were evaluated by obtaining the 2D and M-mode echocardiographic images (Figure 2A). Echocardiographic analysis of two-, four-, and six-month-old csPIMT^−/−^ mice revealed increased left ventricular end-diastolic internal dimension (LVID-d), decreased fractional shortening and also decreased ejection fraction (see Figure 2B for quantification of these changes). At six months of age, the contractility of csPIMT^−/−^ mouse heart was diminished with a fractional shortening of 18% vs. 67% for littermate controls. Likewise, the ejection fraction in four- and six-month-old csPIMT^−/−^ mouse was 57% and 41%, respectively vs. 79% and 73%, respectively, for the floxed littermate controls. These values suggest poor contractility of PIMT null hearts and support the conclusion that PIMT deficient mice die of heart failure.

### 2.4. Global RNA Sequence Analysis of csPIMT^−/−^ Hearts Suggests that Loss of PIMT Affects Multiple Pathways That Are Critical for Heart Function

The structural and functional changes observed so far in the heart of csPIMT^−/−^ mice prompted us to evaluate the alterations in myocardial gene expression. First, we carried out expression profile analysis of PIMT^−/−^ heart tissue for two- and six-month-old mice using the RNA-seq approach to obtain a global view of changes in gene expression. Heart RNA samples from five controls and five csPIMT^−/−^ mice (two- and six-month-old) were pooled then subjected to RNA-seq protocol [9]. For the two-month-old mice, the RNA analysis identified a total of 708 genes with greater than two-fold expression difference between control and PIMT^−/−^ heart RNA samples. Of these, 635 genes showed decreased expression, whereas expression of the rest of the genes was elevated. The down- and up-regulated genes at two months time point are presented in Supplemental section (Tables S1 and S2, respectively). Similarly, for the six-month-old mice, 600 genes showed a greater than two-fold expression difference between control and csPIMT^−/−^ heart RNA samples. These include 417 downregulated genes and 183 upregulated genes; shown in Tables S3 and S4, respectively, in Supplemental section. Some of the down- and upregulated genes classified according to KEGG pathway and their role in some of the pathways related to heart function are shown in Table 1.

The entire list of genes analyzed by RNA-seq that showed significant difference in expression levels at two months and six months has been deposited in Gene Expression Omnibus (GEO number is GSE111862). Overall, these results indicate significant changes in the expression levels of several important genes that would impact on multiple pathways critical for heart function. These include mitochondrial oxidative phosphorylation, energy metabolism, mitophagy, calcium signaling, cardiac muscle contraction, cardiac hypertrophy and myocardial fibrosis. Changes in expression of several of these genes were also confirmed by RT-qPCR. As expected, RT-qPCR analysis validated the changes of gene expression levels observed by RNA-seq analysis (Table 1).

### 2.5. Reduced Expression of Genes Related to Mitochondrial Functions in csPIMT^−/−^ Hearts

**Figure 3:**
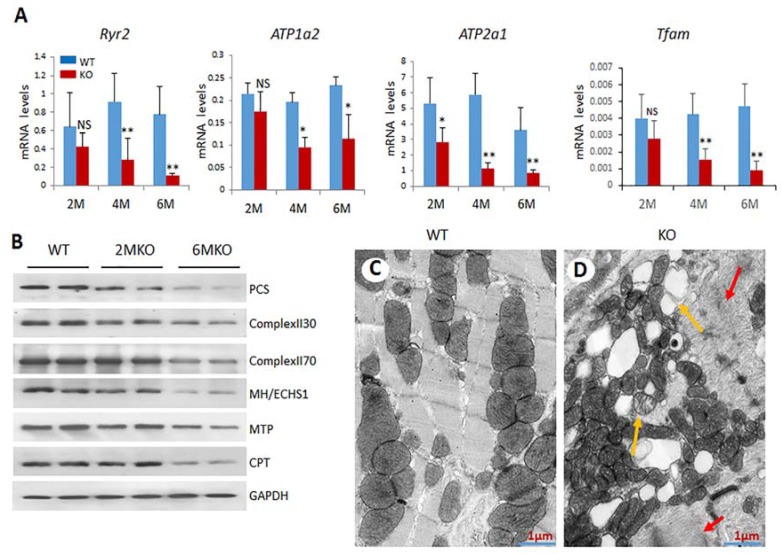
csPIMT^−/−^ hearts show significant mitochondrial damage. (**A**) Quantification of mRNA levels of Atp1a2, Atp2a1, Ryr2 and Tfam genes. Each group was analyzed using 5 different mice (assayed individually) and the values are expressed as the mean ± SD. * *p* < 0.05, ** *p* < 001, NS: not significant; (**B**) Western blot showing protein levels for PCS (palmitoyl-CoA synthetase; 62 Kda), complex II30 and 70, MH/ECHS1 (mitochondrial enoyl-CoA hydratase; 31 Kda), MTP (mitochondrial trifunctional protein; 100 Kda) and CPT1α (carnitine palmitoyltransferase; 88 Kda)). The protein extracts were prepared from 5 hearts pooled together. The protein expression of each gene was normalized to GAPDH. Percent decrease as compared WT controls were as follows: PCS, 67%; complex II30 and 70, 28% and 38%; MTP, 26%, and CPT, 42%; (**C**,**D**) display the electron micrographs of 6-month csPIMT^fl/fl^ and csPIMT^−/−^ mouse hearts. Red arrows in **D** indicate abnormal sarcomeres and H zone absent. Yellow arrows point to lipid droplets and damage in mitochondria.

Genes involved in mitochondrial gene expression and mitochondrial biogenesis are downregulated: Mitochondrial transcription factor A (*Tfam*), is a nuclear encoded gene whose function is to transcribe mitochondrial DNA, and maintain mitochondrial genome copy number [13]. *Tfam* is also necessary for energy generation from oxidative phosphorylation [14]. A 60 to 70% decrease in *Tfam* gene expression was noted in csPIMT^−/−^ mouse heart (Table 1 and Figure 3A) which could explain the reduced population of mitochondria in cardiomyocytes.

**Figure 4:**
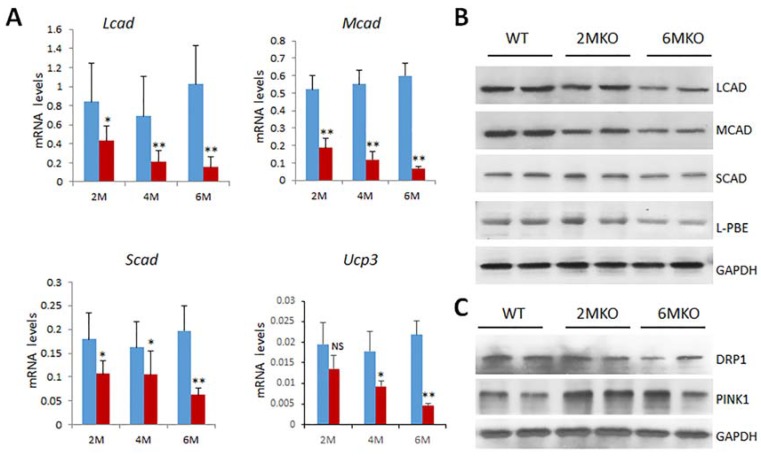
Expression of fatty acid metabolism genes is decreased in csPIMT^−/−^ hearts. (**A**) Quantification of mRNA levels of *Lcad* (Long chain acly-CoA dehydrogenase), *Mcad* (medium-chain acyl-CoA dehydrogenase), *Scad* (short-chain acyl-CoA dehydrogenase) and *Ucp3* (uncoupling protein 3) genes. * *p* < 0.05, ** *p* < 0.01, NS: not significant; (**B**) Western blot showing protein levels of LCAD (47 Kda), MCAD (46 Kda), SCAD (44 Kda) and l-PBE (78 Kda; Enoyl-CoA hydratase /l-3-hydroxyacyl-CoA dehydrogenase). Percent reduction (KO vs. WT) for LCAD, 40%; MCAD, 39%; SCAD, 45%, and l-PBE, 27% for 6-month time point. Each group was analyzed using 5 different mice and the values were expressed as the mean ± SD; (**C**) Western blot showing the protein levels of DRP1 (78 Kda) and PINK1 (60 Kda). Details of same as in (**A**). See Materials and Methods for antibody sources and dilutions.

Genes involved in oxidative phosphorylation and respiratory chain complexes and fatty acid β-oxidation pathway are expressed at lower levels: In mitochondria, ATP is generated in inner mitochondrial membrane by five respiratory complexes (Complexes I, II, III, IV and V) through coupled electron transport and oxidative phosphorylation [15,16]. Reduced expression of any one of the genes related to these subunits would affect oxidative phosphorylation and ATP production. Examples of genes whose expression levels decreased include Ndufs4, Ndufaf4, Ndufaf5, Cox7b and Cox10, Sucla2 and Sdha [17], (Table 1). Expression of the mitochondrial genes were also assayed using Western blots which showed a significant reduction in the protein levels for Complex II, which catalyzes three out of the four steps in β-oxidation [17] (Figure 3B and Figure 4).

Expression of mitochondrial calcium homeostasis related genes is reduced: RNA-seq data of csPIMT^−/−^ hearts, which, in several cases were confirmed by RT-qPCR show significant changes in the expression levels of key genes, namely *Atp2b1*, *Atp2a1*, *Ryr2*, *Cacnb1*, and *Pde1c* that are involved in calcium signaling pathway and cardiac muscle contraction (Table 1). These changes in gene expression could contribute to the development of DCM and are consistent with the echocardiographic observations shown in Figure 2, which indicated poor contractility of PIMT null hearts.

Electron microscopic analysis of csPIMT^−/−^ heart reveal structural damage to mitochondria: Gene expression data suggest dramatic changes in mitochondrial functions in csPIMT^−/−^ mouse heart. To further analyze the damage occurred in mitochondria of csPIMT^−/−^ myocardial cells, we carried out electron microscopic analysis of six-month-old csPIMT^−/−^ heart tissue. Results shown in Figure 3D indicate the presence of lipid vacuoles of differing sizes in cardiomyocytes (Figure 3D). Some mitochondria contained lipid droplets and membranous swirls (*yellow arrows*). Irregularities in Z band pattern were also noted (*red arrows*). These observations combined with the changes in gene expressions described above strongly argues that loss of PIMT leads to damage in mitochondria.

csPIMT^−/−^ cardiomyocytes undergo increased mitophagy. Because we observed severe mitochondrial damage in csPIMT^−/−^ cardiomyocytes, we ascertained whether csPIMT^−/−^ cardiomyocytes display increased mitophagy. Examination of RNA-seq data revealed changes in the expression of several key genes related to mitophagy including *Pink1* (PTEN-induced putative kinase 1), and *Drp1* (dynamin-related protein 1) [18,19,20,21]. These genes play important roles in maintaining mitochondrial homeostasis through complex mechanisms. As shown in Table 1, *Pink1* RNA levels increased between two- to three-fold and Drp1 RNA levels decreased two-fold. The changes in the expression of these genes were also confirmed by Western blots (Figure 4C). In agreement with RNA data, PINK levels increased at least two-fold whereas DRP1 levels decreased two-fold. To sum up, these results suggest that csPIMT^−/−^ heart cells undergo increased mitophagy, and also possibly decreased fission due to increased mitochondrial damage.

Genes involved in the β-oxidation process are expressed at lower levels: Heart muscle cells contract constantly in a coordinated fashion. Therefore, to maintain its contractile function, heart cells must receive constant supply of metabolic substrates to generate ATP. The major source (about 70%) of the energy for cardiac muscle cells come from fatty acids, especially long chain fatty acids [22]. The remainder of the energy in myocardial cells is derived from glucose and lactose [22]. β-Oxidation, a catabolic process by which fatty acid molecules are oxidized, is primarily facilitated by an enzyme complex (mitochondrial trifunctional protein, MTP) that is associated with the inner mitochondrial membrane. Therefore, we assessed the expression levels of genes related to fatty acid oxidation. Data presented in Table 1 show a reduced expression of several genes involved in fatty acid oxidation including *Ppar*α, *Pgc1α*, *Mtp*, *Mcad*, *Ucp*3 and *Abcc9* [23,24]. Decreased expression of several of these genes was also confirmed at the protein level by analyzing key mitochondrial and peroxisomal fatty acid β-oxidation enzymes. Western blots from total cell extracts derived from two- and six-month-old csPIMT^−/−^ hearts along with that of csPIMT^fl/fl^ hearts reveal that protein levels for mitochondrial enoyl-CoA hydratase (ECHS1), MTP, MCAD, SCAD and peroxisomal EHHADH/L-PBE are decreased two- to five-fold as compared to that of control heart extracts (Figure 3B and Figure 4B). These data are consistent with the decreased RNA levels shown in Table 1.

### 2.6. csPIMT^−/−^ Mice Develop Cardiac Fibrosis

**Figure 5:**
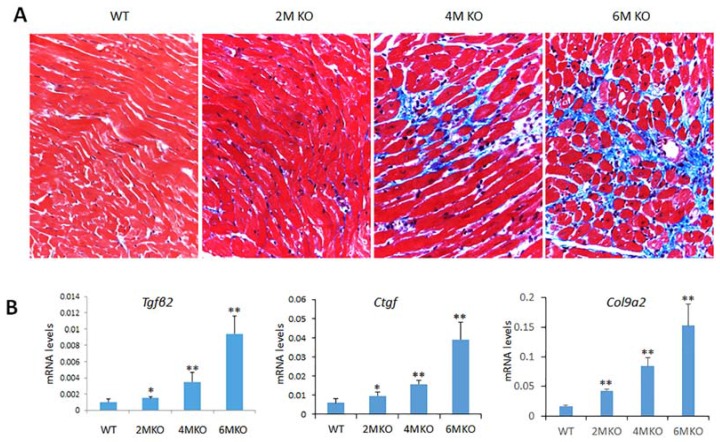
Myocardial fibrosis in csPIMT^−/−^ mouse hearts. (**A**) Images of Masson trichrome staining patterns of a representative PIMT^fl/fl^ and csPIMT^−/−^ hearts of 2, 4 and 6 months are shown (magnification 400×). Note the intensely stained (blue color) interstitial fibrous strands in 6-month-old csPIMT^−/−^ hearts; (**B**) Quantification of mRNA levels for *Tgfβ2*, *Ctgf* and *Col9a2* in csPIMT^fl/fl^ and csPIMT^−/−^ hearts of 2, 4 and 6 months of age. mRNA levels were quantified by RT-qPCR assays. Each group was analyzed using 5 different mice and the values were expressed as the mean ± SD. * *p* < 0.05, ** *p* < 0.01.

Cardiac fibrosis is an important complication in all types of heart diseases including DCM and it is associated with excessive accumulation of extra cellular matrix. To determine whether DCM in csPIMT^−/−^ heart is associated with cardiac fibrosis, we examined the heart tissue for fibrosis by staining paraffin sections of heart with Masson’s trichrome staining. Figure 5A shows significant fibrosis in csPIMT^−/−^ heart as compared to the normal hearts at six months of age. Gene expression analysis supported this observation. At six months of age both RNA-seq and RT-qPCR data showed elevated levels of *Tgfβ2*, *Ctgf*, *Col9a2*, *Mmp3* and *Timp1* RNAs that stimulate signaling mechanisms involved in the regulation of extracellular matrix and promote fibrosis [25,26] (Table 1 and Figure 5B).

### 2.7. Genes Related to Glucose Metabolism Are Downregulated in csPIMT^−/−^ Heart Leading to Glycogen Storage

As stated above, glucose and lactose also serve as significant energy source for myocardial cells. Glucose transporters (GLUT) are a family of proteins which mediate entry of glucose into cells [27,28]. Of these, GLUT4 is the most abundant glucose transporter in heart [29]. We observed a three-fold reduced expression of *Glut4* and hexokinase 2 (*Hk2*, ref [30]) in csPIMT^−/−^ heart as compared to that of PIMT^fl/fl^ heart (Table 1) that potentially could reduce uptake of glucose by myocardial cells (*Glut4*), phosphorylation of glucose (*Hk2*) and curtail the energy source from glucose and lactose for heart cells (Table 1). A likely consequence of reduced expression of these genes is that glucose is not properly utilized in csPIMT^−/−^ myocardial cells and stored as glycogen. We also noted significant reduction in the *Pdk4* mRNA level in the myocardium of csPIMT^−/−^ mice (Figure S1). This enzyme plays a key role in the regulation of glucose and fatty acid metabolism via phosphorylation [31]. Glucokinase (*Gck1,2*) provides G6P for the synthesis of glycogen [32]. GK mRNA level is increased in PIMT^−/−^ heart, as compared to that of PIMT^fl/fl^ heart (Figure S1).

### 2.8. Tamoxifen-Inducible Heart-Specific Cre-Recombinase to Disrupt PIMT Gene (TmcsPIMT^−/−^) in Adult Mouse

**Figure 6:**
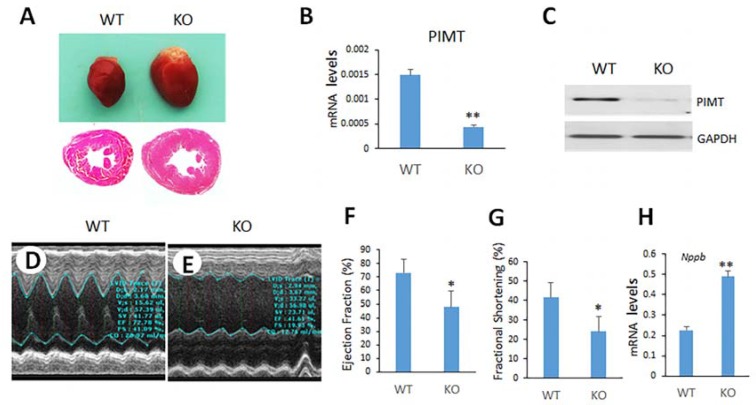
Tamoxifen-inducible cardiac-specific disruption of PIMT (TmcsPIMT^−/−^) in adult mice causes dilated cardiomyopathy. Mice were killed 14 days after first tamoxifen injection in the experiments. (**A**) Representative photographs of adult hearts after tamoxifen-inducible heart-specific Cre mediated PIMT deletion. It is evident that TmcsPIMT^−/−^ mouse heart is bigger than that of TmcsPIMT^fl/fl^ mouse. Lower panel in (**A**) shows H&E cross sections of TmcsPIMT^−/−^ and TmcsPIMT^fl/fl^ hearts; (**B**) Relative PIMT mRNA expression in TmcsPIMT^fl/fl^ (WT) and TmcsPIMT^−/−^ (KO) mouse heart. (**C**) Western blot analysis of PIMT in TmcsPIMT^fl/fl^ and TmcsPIMT^−/−^ hearts. Total proteins from the heart tissues of appropriate mice were prepared as described (see Materials and Methods). They were then Western immunoblotted and probed with an anti-PIMT antibody (Bethyl IHC-00467, 1:1000); (**D**,**E**) Representative profiles of M-mode echocardiographic analyses of TmcsPIMT^−/−^ and littermate control mice; (**F**,**G**) represent ejection fraction and fractional shortening respectively. Data were derived from (**D**,**E**); (**H**) Relative mRNA levels of BNP (*Nppb*) in TmcsPIMT^fl/fl^ and TmcsPIMT^−/−^ mouse hearts. The day of initial injection of Tamoxifen was counted as day 1. Results are expressed as the mean ± SD. * *p* < 0.05, ** *p* < 0.01.

To further validate the findings that lack of PIMT expression is solely responsible for the heart abnormalities and associated heart failure observed in csPIMT^−/−^ mice, we used tamoxifen-inducible heart-specific Cre (Myh6-MCM)/PIMT^fl/fl^ mouse model (TmcsPIMT^−/−^). The tamoxifen-inducible gene knockout approach has clear advantages in that expression of a selected gene can be ablated in adult mice, as necessary, in a tissue-specific manner [33]. The Myh6-MCM/PIMT^fl/f^ mice were given daily intraperitoneal injection of tamoxifen for five days. By 14 days after the first tamoxifen injection, the size of the heart increased dramatically as compared to that of littermate controls (Figure 6A, upper panel). Figure 6A also shows (lower panel) dilatation of the left ventricular chambers with thinning of the walls. PIMT RNA levels become almost non-detectable in heart (Figure 6B). PIMT expression was also evaluated using Western blot method. Figure 6C shows that PIMT expression was negligible in TmcsPIMT^−/−^ hearts as compared to control hearts. Immunostaining of TmcsPIMT^−/−^ heart tissue confirmed the absence of PIMT in nuclei of TmcsPIMT^−/−^ mouse cardiomyocytes Echocardiographic analysis of TmcsPIMT^−/−^ mice heart revealed increased left ventricular end-diastolic internal dimension (LVID-d), decreased fractional shortening and also decreased ejection fraction (Figure 6D,E). The contractility of TmcsPIMT^−/−^ mouse heart was diminished with the ejection fraction in TmcsPIMT^−/−^ mouse was 45% vs. 78% for floxed littermate controls (Figure 6F). Likewise, a fractional shortening of 21% vs. 43% for littermate controls was also observed (Figure 6G). These values suggest poor contractility of PIMT null hearts and support the conclusion that PIMT deficient mice die of DCM. Accordingly, the mRNA levels of heart failure indicator BNP were significantly elevated in TmcsPIMT^−/−^ mouse heart (Figure 6H).

We also examined whether TmcsPIMT^−/−^ hearts develop cardiac fibrosis. As shown in Figure S2, Masson Trichrome staining indicated significant cardiac fibrosis in PIMT null hearts in Tamoxifen inducible model (shown by arrows) which is in agreement with the development of cardiac fibrosis in csPIMT^−/−^ hearts. There was also significant increase in the RNA levels of *Ctgf* and Tgfβ2 genes (Figure S2C,D).

## 3. Discussion

The molecular mechanisms that lead to the development of DCM are not well understood. We now report the role played by PIMT in mouse heart functions. PIMT, an RNA binding protein with methyl transferase activity, participates in the formation of 2,2,7-trimethylguanosine cap structures of small nuclear and nucleolar RNAs that are important in RNA splicing [1]. Furthermore, PIMT interacts with nuclear receptor coactivators NCOA6, p300/CBP histone acetyltransferases, and the MED1 subunit of the Mediator complex and these interactions appear to influence energy metabolism in heart [8]. Mice with germ line deletion of *Pimt* gene, manifest early embryonic lethality by affecting development during preimplantation stage [11]. These and other results suggest that PIMT has the potential to control metabolic pathways at the chromatin level by influencing fatty acid oxidation and gluconeogenesis-related genes on its own merit and in concert with other transcription factors.

The results presented in this paper clearly demonstrate that PIMT is an essential gene for normal heart function and that heart-specific ablation of PIMT results in DCM (Figure 2 and Figure 6). Cardiomyocyte-specific conditional PIMT deleted mice (csPIMT^−/−^ mice) died by 7.5 months of age (this manuscript) whereas csMed1^−/−^ mice died at age of one month [9]. It is also worth noting that deletion of the Mediator subunit genes including *Med1*, *Med12* or *Med30* in heart is more damaging in causing DCM [9,34,35,36]. It is not surprising because Med1 is necessary for the completion of transcriptional signaling of PPAR subfamily nuclear receptors [3].

The heart sections of two-month-old csPIMT^−/−^ mice showed detectable thinning of the ventricular walls with considerable ventricular enlargement (Figure 1). That the two-month-old csPIMT^−/−^ hearts suffer with DCM is supported by the data showing elevated levels of mRNAs coding for the BNP and ANP proteins. Both BNP and ANP levels increase during heart failure as ventricular cells secrete both these peptides in response to left ventricular dysfunction [12]. Left ventricular dilation increases progressively in csPIMT^−/−^ hearts as evidenced by the images of H&E stained heart cross sections and sustained increase of ANP and BNP RNA levels (Figure 1). Other evidence including diminished contractility of csPIMT^−/−^ hearts, loss of structural integrity of mitochondria and reduced expression of most of the genes related to oxidative phosphorylation and the fatty acid β-oxidation. Evidence supports the assertion that mitochondrial damage significantly contributes to the development of DCM and myocardial dysfunction. Cardiac myocytes are a type of muscle cells that contract and expand continuously, and this contractility is dependent on the constant supply of ATP generated by mitochondria through a complex interaction between oxidative phosphorylation and electron transport chain systems and both mitochondrial and peroxisomal fatty acid β-oxidation systems. Our gene expression analysis showed that the majority of the genes related to both these β-oxidation systems are expressed at lower levels in csPIMT^−/−^ hearts at four and six months of age. For example, expression of several genes related to mitochondrial complexes I to IV and the mitochondrial uncoupling protein UCP3 decreased by about two- to five-fold. Similarly, expression of genes such as *Ppar*α, *Pargc1a*, *Acadm* that are involved in energy homeostasis is also reduced about two- to six-fold. Another important gene *Tfam*, involved in mitochondrial DNA replication and transcription is also expressed at six-fold reduced levels that correlates with decreased mitochondrial copy number in csPIMT^−/−^ hearts. TFAM is a multifunctional transcriptional factor that is critical for the mitochondrial DNA transcription and maintenance of mitochondrial genome copy number [13]. The mitochondria of csPIMT^−/−^ heart cells also suffer structural damage as evidenced by the presence of lipid droplets and membranous swirls and irregularities in Z band pattern (Figure 3). In addition, evidence indicates that csPIMT^−/−^ cardiomyocytes display increased mitophagy. In summary, mitochondria of csPIMT^−/−^ hearts are unable to perform their normal functions and thus contribute to DCM and heart failure.

The role played by the peroxisomal and mitochondrial β-oxidation in the pathogenesis of cardiomyocyte and mitochondrial damage leading to the development of DCM is that both inhibition and profound elevation of fatty acid β oxidation can be pathogenic [37]. Diminished fatty acid β-oxidation can occur in the absence of PPARα and this causes toxic lipid injury due to un-metabolized very long chain fatty acids and fatty acyl CoAs and other intermediate metabolic products [37]. Likewise, excess fatty acid β-oxidation resulting from PPARα activation can also be deleterious [37,38].

Proper transport of calcium in and out of cardiac myocytes and coordination between calcium channel function and ATP production are critical for normal heart functions. Defects in calcium regulation and energy production are hallmarks of heart failure [39]. Global RNA analysis of csPIMT^−/−^ heart tissue showed downregulation of several genes related to calcium channel structure and function, cardiac muscle contraction and calcium homeostasis. For example, *Atp2a2* (also known as *Serca-2a*) which encodes Ca2β ATPase isoform 2a protein and *Cacna1h* encodes a structural protein of voltage gated calcium channel are involved in calcium mediated changes in cardiomyocyte contractility [39]. Similarly, ryanodine receptor 2 gene (*Ryr2*), which encodes Ryr2 protein initiates cardiac muscle contraction by calcium channeling [29]. *Ryr2* regulates mitochondrial Ca^2+^ and ATP levels as well as a cascade of transcription factors that modulate metabolism and survival [39,40]. Overall, these results suggest that calcium regulation is defective in myocardial cells of csPIMT^−/−^ hearts owing to reduced expression of the relevant genes described above. Thus, PIMT along with Med1 and Med12 contribute to the regulation of calcium handling genes [9,36].

We also observed significant myocardial fibrosis in csPIMT^−/−^ heart at six months of age. In normal heart, the fibroblasts form a network throughout myocardium and contribute in part to the mechanical and structural maintenance of heart [41]. When there is cardiac injury, myocardial fibroblasts are activated due to cytokine and neurohumoral factors released by the heart tissue which deregulate the extracellular matrix leading to the development of fibrosis [26]. The fibrosis process involves activation of a number of genes related to formation of extracellular matrix including *Tgfβ*, connective tissue growth factor (*Ctgf*), matrix metalloprotease *Mmp3*, alpha 2 type IX collagen (*Col9a2*), and the tissue inhibitor of metalloproteinase-1 Timp1 [26]. Our RNAseq and RT-qPCR data confirmed the upregulation of these genes beginning two months after birth, with the expression levels ranging from 1.5- to eight-fold depending on the type of the gene. Sustained upregulation of these genes was observed at the age of six months that could explain the development of the cardiac fibrosis in csPIMT^−/−^ hearts.

**Figure 7:**
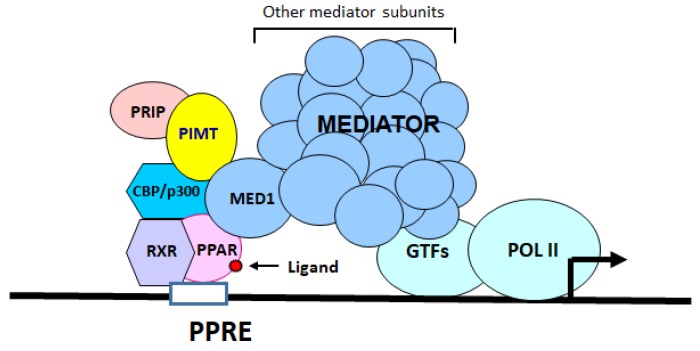
A model showing the interactions of PIMT with PRIP/NCOA6, histone acetyl transferases p300/CBP, and MED1 of the Mediator complex in the regulation of transcription of PPAR regulated genes. Note that in addition to Med1 subunit of the Mediator complex MED30 and MED12 also modulate genes involved in energy metabolism. See Discussion for further details. See Discussion for further details.

At present, we do not know the molecular mechanisms by which PIMT affects gene expression except that it occurs at the chromatin level. We showed earlier that PIMT physically interacts with NCOA6, p300/CBP, and MED1 to transcriptionally stimulate reporter genes [8]. Based on these observations, we propose a model in which PIMT forms a complex with NCOA6 and cooperates with histone acetyl transferases p300/CBP, MED1 and perhaps with other unknown chromatin factors to affect transcription of a group of genes specific for cardiac functions [8] (see Figure 7). This model at least in part can explain the broad effects we observed about PIMT deletion in PIMT^−/−^ hearts. However, additional studies are needed to address many of the issues raised in this study.

In summary, we have for the first time, reported here an essential function for PIMT gene in heart function and consequences of ablation of PIMT gene in heart including the development of DCM and heart failure. Thus, PIMT is a member of a growing list of essential genes that are critical for heart function and understanding the PIMT functions in heart will aid in the efforts to develop novel drugs and other therapeutic strategies in the management of heart failure.

## 4. Materials and Methods

### 4.1. Animals

PIMT conditional knock-out mice were generated using the two-loxP, two-frt recombination system [42]. PIMT^fl/fl^ mice were crossbred with cardiac α-myosin heavy chain promoter driven Cre (α-MyHC-Cre) transgenic mice [11] to generate cardiomyocyte-specific PIMT null mice (csPIMT^−/−^) with deletion of exons 3 and 4 of *PIMT* gene commencing during late embryonic period. PCR genotyping was performed using the primers P4: 5′-CTGCATGTATGAATCTTGGGAG-3′, P5: 5′-GCATCAAGAATATACAGAACAGAGA CTC-3′ and P6: 5′-CTCCTTCCTTCTGTACCTCTGTAGC-3′. Primers P6/P5 yielded a 376 bp Wild-type PIMT allele in PIMT^+/+^ mice; primers P4/P5 yielded a 298 bp PIMT^fl/fl^ allele. *Cre*-specific primers used included: 5′-AGGTGTAGAGAAGGCACTCAGC-3′ and 5′-CTAATCGCCATCTTCCAGCAGG-3′.

To generate mice with tamoxifen-inducible heart-specific PIMT deletion (TmcsPIMT^−/−^), PIMT^fl/fl^ mice were cross-bred with Myh6-MCM (tamoxifen-inducible heart-specific Cre) transgenic mice purchased from the Jackson Laboratory [33]. TmcsPIMT^−/−^ mice and their littermate controls were administered tamoxifen intraperitoneally at seven weeks of age at a daily dose of 65 mg/kg body weight for five days and then killed at selected intervals. Survival curves were obtained by following 36 csPIMT^−/−^ mice and the same number of csPIMT^fl/fl^ genotype. The criteria used for animal euthanasia were as listed previously [5], and included absence of food and water consumption, diminished or absence of mobility, absence of heart beat and respiratory movement. Pentobarbital was injected intraperitoneally at the dose of 150 mg/kg body weight to minimize suffering. Animals had access to food and water ad libitum and maintained on a 12-h light-dark cycle. All procedures were performed in accordance with the National Institutes of Health Guide for Care and Use of Laboratory Animals. The animal protocols were reviewed and approved by the Institutional Animal Care and Use Committee of Northwestern University (protocol number 2013–3198, 1 July 2013).

### 4.2. Echocardiography

Echocardiography was performed as described previously using a VisualSonics Vevo 770 high-resolution noninvasive transthoracic imaging system with a 30 MHz scanhead [5]. Short- and long-axis parasternal views were used to obtain 2D and M-mode images which facilitated examination of the septum, posterior wall and left ventricular outflow tract. We recorded at least eight independent cardiac cycles per experiment.

### 4.3. Histological Analysis

Heart tissues from csPIMT^−/−^ and PIMT^fl/fl^ and also from TmcsPIMT^−/−^ and the corresponding control mice were fixed in 4% paraformaldehyde for 48 h and processed for embedding in paraffin. Sections, 4-μm thick, were stained with hematoxylin and eosin (H&E). Immunohistochemical localization of PIMT was carried out using anti-PIMT antibody (catalog number IHC-00467, Bethyl, Montgomery, TX, USA). Masson’s trichrome staining was used for the visualization of cardiac fibrosis. Heart specimens were also embedded in O.C.T. compound (Tissue-TeK, Torrance City, CA, USA), and 6-um thick sections were stained with Oil Red O for the visualization of neutral lipid.

### 4.4. Electron Microscopy

Heart tissue samples obtained from the left ventricle were fixed overnight at 4 °C with 3% glutaraldehyde in sodium cacodylate buffer. The tissue was then post-fixed in 1% osmium tetroxide in cacodylate buffer (pH 7.4) for 2 h at 4 °C and embedded in Epon [9]. Ultra-thin sections were cut with a Leica UC6 ultramicrotome and examined with a FEI Tecnai Spirit transmission electron microscope (FEI, Hilsboro City, OR, USA).

### 4.5. Library Construction and Sequencing

Library construction and sequencing were carried out at the Genomics Core facility of University of Chicago. To generate single-end 50 bp (SR50) RNA sequencing libraries, RNA quality and quantity were determined with Agilent Bioanalyzer 2100, selecting RNA integrity numbers (RIN) of >7 and quantities of 100 nanograms or more per sample. Directional mRNA libraries were generated using Illumina TruSeq mRNA Sample Preparation Kits (Illumina, San Diego, CA, USA). Briefly, polyadenylated mRNAs were captured from total RNA using oligo-dT selection and then converted to cDNA by reverse transcription. They were then ligated to Illumina sequencing adapters containing unique barcode sequences. These were then amplified by PCR and the resulting cDNA libraries quantified using RT-RT-qPCR. Finally, equimolar concentrations of ach cDNA library were pooled and sequenced on the Illumina HiSeq2500 (Illumina, San Diego, CA, USA).

### 4.6. Transcriptome Analysis

The quality of DNA reads, in fastq format, was evaluated using FastQC. Adapters were trimmed and reads of poor quality or aligning to rRNA sequences were filtered. The cleaned reads were aligned to the *Mus musculus* genome (mm10) using STAR [43]. Read counts for each gene were calculated using htseq-count [44] in conjunction with a gene annotation file for mm10 obtained from UCSC (University of California Santa Cruz; http://genome.ucsc.edu). Differential expression was determined using DESeq2 [45]. The cutoff for determining significantly differentially expressed genes was an FDR-adjusted *p*-value less than 0.05. A pathway analysis was performed on both upregulated and downregulated gene lists using GeneCoDis [46,47].

### 4.7. Quantitative Real-Time PCR

Total RNA was extracted from the csPIMT^−/−^ and TmcPIMT^−/−^ and the corresponding control mice using TRIzol^®^ reagent (Life Technology, Carlsbad, CA, USA). RNA was further purified using Qiagen RNeasy columns (Life Technology, Carlsbad, CA, USA). cDNA was prepared with 2 μg of total RNA using SuperScript VILO First-Strand Synthesis System (Life Technology, Carlsbad, CA, USA). Expression of specific genes was verified using SYBR Green (Life Technologies) in triplicates and normalized with 18S ribosomal RNA. Each PCR reaction contained 1 μL (100 pmol) of forward and reverse primers and 10 μL of 2× SYBR Green PCR Master Mix to make a final volume of 20 μL. The reaction was run by using an ABI 7300 (Applied Biosystems, Foster City, CA, USA). The relative gene expression changes were measured using the comparative *C*_t_ method, *X* = 2^−ΔΔ*C*t^. Sequences of all primers are shown in Table S5.

### 4.8. Western Blot Analysis

Total proteins were extracted from the heart tissue of csPIMT^−/−^ mice and corresponding littermates and subjected to 4–20% SDS-PAGE. Samples were analyzed in duplicates for each time point. Protein extracts were prepared from pooled samples using five animals. Same pooled hearts were used for protein extracts and for RT-qPCR assays, as well as for RNA-seq analysis. They were then transferred to a nitrocellulose membrane (Invitrogen). Immunoblotting was performed using relevant antibodies as described with GAPDH as loading control. The protein bands were developed with an enhanced chemiluminescence substrate. Quantification of blots was performed using ImageJ software (NIH). Sources of antibodies and dilutions: Complex II30, Invitrogen cat# 459230; ComplexII70, Invitrogen cat#459200; DRP1, Cell Signaling, cat#8570; Pink1, Cell Signaling cat#6946. All antibodies mentioned above were diluted 1:1000. GAPDH, Cell Signaling cat# 5174, dilution 1:1500. PCS, MH, MTP, CPT, LCAD, MCAD, SCAD, L-PBE antibodies (dilution 1:2000) are rabbit polyclonal antibodies, kind gifts of Dr. T. Hashimoto, Department of Pediatrics, Gifu University School of Medicine, Japan.

### 4.9. Mitochondrial DNA Content

To determine the mitochondrial DNA copy number, total DNA from heart tissue was first isolated. The quantity of nuclear-encoded 18S ribosomal RNA (rRNA) and the mitochondrial encoded gene cytochrome c oxidase subunit 1 (CO1) were estimated by RT-qPCR. Mitochondrial DNA copy number was expressed as the ratio of CO1 to 18S rRNA as described [9,48].

### 4.10. Statistical Analysis

Student’s *t* test was used to determine whether the sample was significantly different from the control. Differences were considered statistically significant at *p* < 0.05, while *p* < 0.01 represented more significant change.

## Figures and Tables

**Table 1:** Gene expression changes in csPIMT^−/−^ relative to PIMT^fl/fl^ mouse heart.

Function	Gene	KO/WT	KO/WT	KO/WT	KO/WT
		2 M, qPCR	2 M RNA-Seq	6 M, qPCR	6 M, RNA-Seq
OXPHOS					
	*Ndufaf4*	0.35 *	0.21	0.18 **	0.20
	*Ndufaf5*	0.29 *	0.33	0.21 **	0.17
	*Ndufs4*	0.51 *	0.42	0.27 **	0.24
	*Cox7b*	0.32 *	0.27	0.36 *	0.41
	*Cox10*	0.59	0.27	0.25 **	0.28
	*Sdha*	0.63	0.52	0.41 *	0.37
	*Sucla2*	0.24 *	0.12	0.12 **	0.08
Energy metabolism/fatty acid					
	*Pparα*	0.54	0.66	0.49	0.57
	*Ppargc1a*	0.51	0.36	0.55	0.45
	*Acadm*	0.36 *	0.39	0.12 **	0.27
	*Ucp3*	0.63	0.61	0.15 **	0.24
	*Abcc9*	0.61	0.67	0.16 *	0.49
Glucose metabolism					
	*Gck(GK)*	3.93 *	1.97	2.46 *	3.87
	*Pck1*	1.88	4.23	0.51	0.63
	*Pdk4*	0.38 *	0.37	0.24 *	0.99
	*HK2*	0.74	0.97	0.45 *	0.83
	*Glut4*	0.59	0.92	0.36 *	0.78
Transcription factor, coactivator					
	*Med1*	0.64	0.51	0.66	0.48
	*NcoA6*	0.69	0.66	0.57	0.62
	*Tfam*	0.47	0.25	0.19 **	0.48
Mitophagy/mitochondria fission					
	*Pink1*	2.16 *	1.93	1.57	1.65
	*Drp1*	0.58	0.41	0.39 *	0.46
Cardiomyopathy/Fibrosis					
	*Tgfb2*	0.66	0.91	9.41 **	5.23
	*Ctgf*	1.16	2.83	6.42 **	7.71
	*Col9a2*	3.24 *	6.42	8.33 **	26.06
	*Fgf6*	1.86	2.37	10.92 **	17.65
	*Fgf21*	2.35 *	3.08	5.18 **	6.22
	*Mmp3*	3.92 *	2.37	10.86 **	7.61
	*Timp1*	4.58 **	4.97	4.63 **	6.18
Hypertrophy/dilation					
	*Atf3*	1.19	1.57	3.91 *	3.02
	*Ace*	2.69 *	2.23	3.78 *	6.48
	*Wisp2*	4.72 **	3.62	11.61 **	8.74
	*Thbs4*	5.91 **	6.68	9.34 **	8.56
Calcium homeostasis and signaling pathway					
	*Atp2b1*	0.34 *	0.22	0.31 *	0.25
	*Atp2a1*	0.53	0.52	0.23 *	0.43
	*Ryr2*	0.65	0.53	0.14 **	0.54
	*Map3k6*	2.14	3.07	3.01 *	8.01
	*Cacnb1*	2.49 *	1.98	3.72 *	5.12
	*Pde1c*	0.38	0.21	0.21 **	0.14
	*Cacna1h*	0.29 *	0.23	0.19 **	0.01
	*Mapk8*	0.27 *	0.21	0.33 *	0.18

* *p* < 0.05, ** *p* < 0.01. KO, csPIMT^−/−^; WT, PIMT ^fl/fl^; M, months.

## Supplementary Materials

Supplementary materials can be found at http://www.mdpi.com/1422-0067/19/5/1485/s1.
