# Supplementary material for: PIMT/NCOA6IP Deletion in the Mouse Heart Causes Delayed Cardiomyopathy Attributable to Perturbation in Energy Metabolism
Source: Int J Mol Sci. 2018 May 16;19(5):1485. doi: 10.3390/ijms19051485 (PMC5983783; doi:10.3390/ijms19051485)
Supplement: Supplementary file 1 [file ijms-19-01485-s001.zip › ijms-306114-supplementary/Figures S1&2.pdf]

Supplementary data:

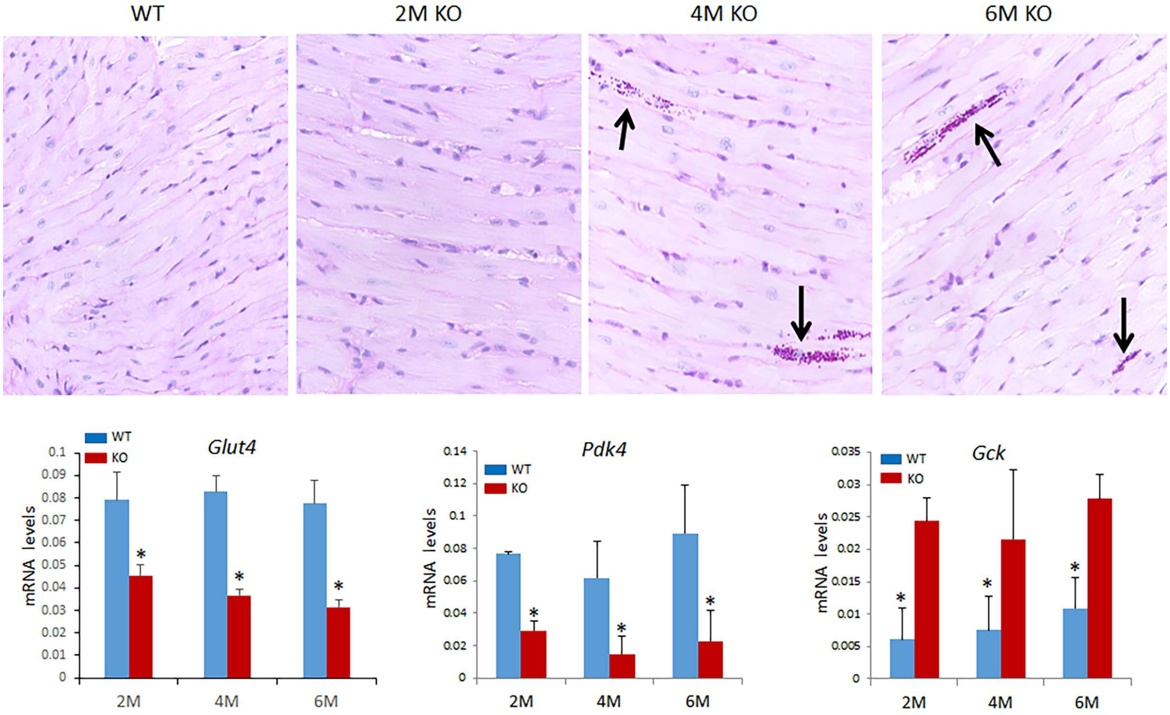

Figure S1. Glycogen storage in csPIMT$^{-/-}$ mouse hearts. (**A**) Images of PAS staining patterns of PIMT$^{fl/fl}$ and csPIMT$^{-/-}$ mouse hearts of 2, 4 and 6 months. Glycogen storage in csPIMT$^{-/-}$ hearts of 4 and 6 months is indicated by black arrows; (**B**) Quantification of mRNA levels for *Glut4, PDK4 and Gck* in PIMT$^{fl/fl}$ and csPIMT$^{-/-}$ mouse hearts. mRNA levels were quantified by RT-qPCR assays. Each group was analyzed using 5 different mic and the values were expressed as the mean ± SD. * $p < 0.05$.

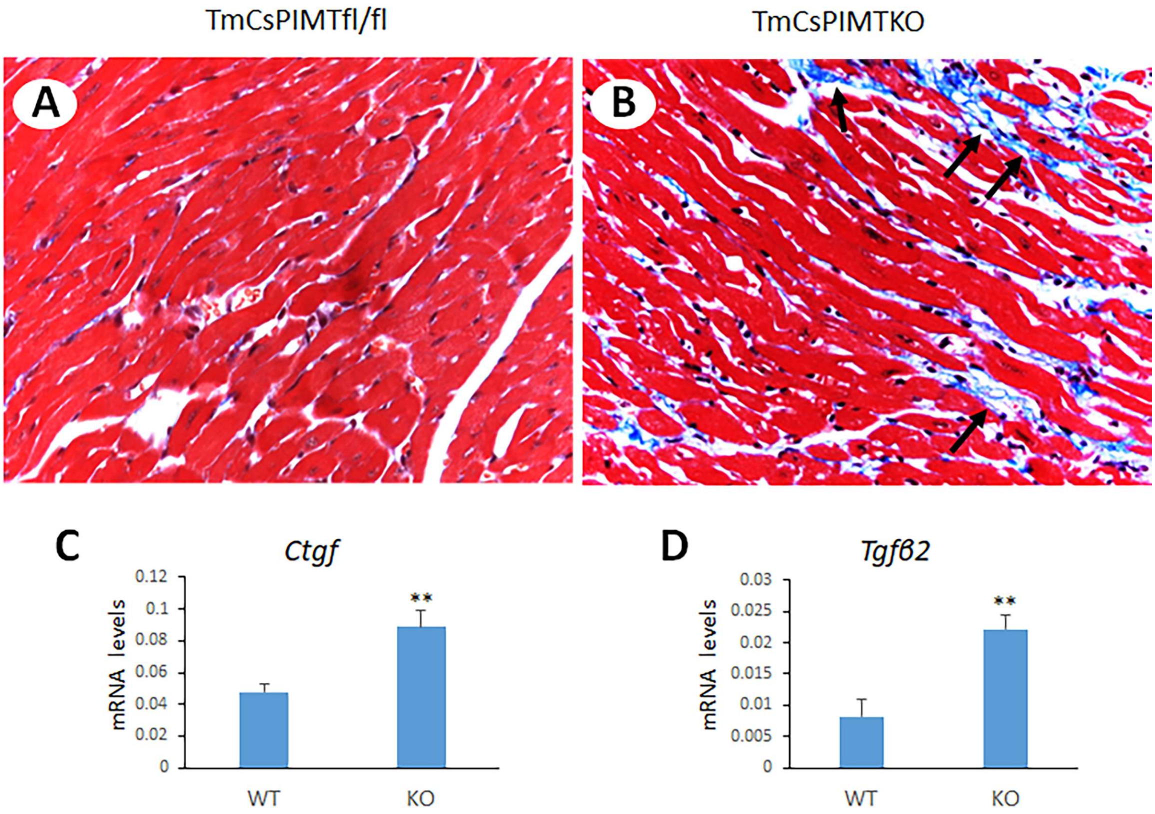

Figure S2. Cardiac fibrosis in TmcsPIMT$^{-/-}$ mouse heart. (**A**,**B**) Masson Trichrome staining images showing fibrosis (intensely stained blue color, shown by black arrow); (**C**,**D**) Quantification of mRNA levels for C*tgf* and *Tgfβ2* in PIMT$^{fl/fl}$ and csPIMT$^{-/-}$ mouse hearts. mRNA levels were quantified by RT-qPCR assays. Each group was analyzed using 5 different mice and values were expressed as the mean ± SD. ** $p < 0.01$.
