# Supplementary material for: PIMT/NCOA6IP Deletion in the Mouse Heart Causes Delayed Cardiomyopathy Attributable to Perturbation in Energy Metabolism
Source: Int J Mol Sci. 2018 May 16;19(5):1485. doi: 10.3390/ijms19051485 (PMC5983783; doi:10.3390/ijms19051485)
Supplement: Supplementary file 1 [file ijms-19-01485-s001.zip › ijms-306114-supplementary/S5 Table Selected primers used for qPCR.docx]

**S5 Table. Selected primers used for qPCR**

| ***Gene name*** | ***Forward primers*** | ***Reverse primers*** |
| --- | --- | --- |
| ***PIMT(Ncoa6IP)*** | ***ATGTGTTGCGAGAAGTGGAAC*** | ***CACTGCTTTTGACATAGTAGCCT*** |
| ***Ndufaf4*** | ***CACCGGAGTCAGTATCCAGAA*** | ***GGTTCAACTTTTACCGGCAAGG*** |
| ***Ndufaf5*** | ***CTGCGAAAGGTCGTTCTTTTG*** | ***CGAAGATGTTTAGGGCTCTGG*** |
| ***Ndufs4*** | ***CTGCCGTTTCCGTCTGTAGAG*** | ***TGTTATTGCGAGCAGGAACAAA*** |
| ***Cox7b*** | ***TTGCCCTTAGCCAAAAACGC*** | ***TCATGGAAACTAGGTGCCCTC*** |
| ***COX10*** | ***TTCCTCAAGCGCATGTATGT*** | ***CTCTAGCCTGCCCTGAAGAC*** |
| ***Sdha*** | ***CTACAAGGGACAGGTGCTGA*** | ***GAGAGAATTTGCTCCAAGCC*** |
| ***Sucla2*** | ***ACCCTTTCGCTGCATGAATAC*** | ***CCTGTGCCTTTATCACAACATCC*** |
| ***PPARα*** | ***GGGCTCCGAGGGCTCTGTCA*** | ***TGCAGCTCCGATCACACTTGTCG*** |
| ***Ppargc1a*** | ***TATGGAGTGACATAGAGTGTGCT*** | ***CCACTTCAATCCACCCAGAAAG*** |
| ***Acadm(MCAD)*** | ***GGCCAGAAGATGTGGATAAC*** | ***GTCGGCTTCCACAATGAA*** |
| ***UCP3*** | ***GGATGTGGTAAAGACCCGAT*** | ***AGGGCACAAATCCTTTGTAGA*** |
| ***Abcc9*** | ***GGATAGTCCAGCGAGTAAATG*** | ***AGAAAGGTCCTCTGTAGGATAA*** |
| ***Med1*** | ***GTGTGGTAATGGATGTGCAGGAC*** | ***AGCTTTCCTCCGAATAGCCCTC*** |
| ***NcoA6*** | ***GAATGTGCCCAACTTGTTACAC*** | ***CCCTTCAATCTGAACGGAGAGAA*** |
| ***Tfam*** | ***CAAGTCAGCTGATGGGTATG*** | ***TGAGCCGAATCATCCTTTG*** |
| ***Tgfb2*** | ***GCGCTACATCGATAGCAAGG*** | ***TAGACGGCACGAAGGTACAG*** |
| ***Ctgf*** | ***CTTCCCGAGAAGGGTCAAG*** | ***CAGTCGGTAGGCAGCTAGG*** |
| ***Col9a2*** | ***TTGTGTCCAACCAACTGTCC*** | ***CTGGATGACCCTTCACTCCT*** |
| ***Fgf6*** | ***CAGGCTCTCGTCTTCTTAGGC*** | ***AATAGCCGCTTTCCCAATTCA*** |
| ***Fgf21*** | ***CTGCTGGGGGTCTACCAAG*** | ***CTGCGCCTACCACTGTTCC*** |
| ***Mmp3*** | ***ACATGGAGACTTTGTCCCTTTTG*** | ***TTGGCTGAGTGGTAGAGTCCC*** |
| ***Timp1*** | ***GCAACTCGGACCTGGTCATAA*** | ***CGGCCCGTGATGAGAAACT*** |
| ***ATF3*** | ***TGACACCCTTTGTCAAGGAA*** | ***GCCTCAGACTTGGTGACTGA*** |
| ***Ace*** | ***GCCTGGGACTTCTACAACCG*** | ***CTCATGGAAGCCAGGGTTGG*** |
| ***Wisp2*** | ***AATACAGGTGCCAGGAAGGT*** | ***AGAAAGTTGGTGTCCTTGGG*** |
| ***Thbs4*** | ***ACGGCTGAACAAAGCCATC*** | ***TTGCTCAGTCTCAGGAGAACC*** |
| ***Atp2b1*** | ***AGATGGAGCTATTGAGAATCGCA*** | ***CCCTGTAACACGGATTTTTCCTT*** |
| ***Atp2a1*** | ***TGTTTGTCCTATTTCGGGGTG*** | ***AATCCGCACAAGCAGGTCTTC*** |
| ***Ryr2*** | ***GATCAGCAGACTGGAAAGAC*** | ***GGCATACACAGAGAGAACAC*** |
| ***Map3k6*** | ***GCCTCTCAGTGTGGTCTACG*** | ***CGTCGCAAAGGGTAGGCTG*** |
| ***Cacnb1*** | ***CCTGCCAGTGGTAATGAAATGA*** | ***CTCACGCTAGTCTTGGCTGA*** |
| ***Pde1c*** | ***CCTCCTTTGCGTCACTTTAAGC*** | ***TCATCTTGATCCGCTGATCCA*** |
| ***Cacna1h*** | ***CGTGACACTGGGCATGTTC*** | ***CCACCATCTTGATAACCATCTCC*** |
| ***Mapk8*** | ***AGCAGAAGCAAACGTGACAAC*** | ***GCTGCACACACTATTCCTTGAG*** |
| ***Gck(GK)*** | ***TGAGCCGGATGCAGAAGGA*** | ***GCAACATCTTTACACTGGCCT*** |
| ***Pck1*** | ***CTGCATAACGGTCTGGACTTC*** | ***CAGCAACTGCCCGTACTCC*** |
| ***Pdk4*** | ***AGGGAGGTCGAGCTGTTCTC*** | ***GGAGTGTTCACTAAGCGGTCA*** |
| ***HK2*** | ***GAACCAGATCTACGCCATTC*** | ***GGGAACGAGAAGGTGAAAC*** |
| ***Glut4*** | ***TCTCCTGCAGCTGATTCT*** | ***CTCTCTCCAACTTCCGTTTC*** |
| ***PINK1*** | ***GGCTTCCGTCTGGAGGATTAT*** | ***AACCTGCCGAGATATTCCACA*** |
| ***Drp1*** | ***CAGGAATTGTTACGGTTCCCTAA*** | ***CCTGAATTAACTTGTCCCGTGA*** |
